# Microtubules as a Critical Target for Arsenic Toxicity in Lung Cells *in Vitro* and *in Vivo*

**DOI:** 10.3390/ijerph9020474

**Published:** 2012-02-01

**Authors:** Yinzhi Zhao, Paul Toselli, Wande Li

**Affiliations:** Department of Biochemistry, Boston University School of Medicine, 72 East Concord Street, Boston, MA 02118, USA; Email: yinzhi@bu.edu (Y.Z.); paulacs@bu.edu (P.T.)

**Keywords:** trivalent arsenic (As^3+^), microtubules (MTs), tubulin, tubulin mRNA, tubulinsulfhydryl groups (-SH), microtubule-associated proteins (MAPs), chromosomal disorientations, metallothionein, taxol

## Abstract

To understand mechanisms for arsenic toxicity in the lung, we examined effects of sodium *m*-arsenite (As^3+^) on microtubule (MT) assembly *in vitro* (0–40 µM), in cultured rat lung fibroblasts (RFL6, 0–20 µM for 24 h) and in the rat animal model (intratracheal instillation of 2.02 mg As/kg body weight, once a week for 5 weeks). As^3+^ induced a dose-dependent disassembly of cellular MTs and enhancement of the free tubulin pool, initiating an autoregulation of tubulin synthesis manifest as inhibition of steady-state mRNA levels of βI-tubulin in dosed lung cells and tissues. Spindle MT injuries by As^3+^ were concomitant with chromosomal disorientations. As^3+^ reduced the binding to tubulin of [^3^H]N-ethylmaleimide (NEM), an -SH group reagent, resulting in inhibition of MT polymerization *in vitro* with bovine brain tubulins which was abolished by addition of dithiothreitol (DTT) suggesting As^3+^ action upon tubulin through -SH groups. In response to As^3+^, cells elevated cellular thiols such as metallothionein. Taxol, a tubulin polymerization agent, antagonized both As^3+^ and NEM induced MT depolymerization. MT–associated proteins (MAPs) essential for the MT stability were markedly suppressed in As^3+^-treated cells. Thus, tubulin sulfhydryls and MAPs are major molecular targets for As^3+^ damage to the lung triggering MT disassembly cascades.

## 1. Introduction

Arsenic (As) is an abundant metalloid element in the Earth’s crust. Its compounds are widely used in industries, agricultures, and medicines [1]. In addition to occupational contact, consumption of contaminated drinking water is a major source of As exposure for humans. Most As in drinking water exists as inorganic forms released from either certain types of rock or man’s past activities [1,2]. An estimated 100–140 million people around the world are suffering from chronic As poisoning [3,4]. Inhaled or ingested As can be rapidly absorbed, enter the bloodstream, and distribute throughout the body [5,6]. The lung is one of the major target organs of As, either from contaminated air or from polluted water, and leading to the development of bronchiectasis, bronchitis, chronic obstructive pulmonary disease (COPD), and malignancies [2,7]. Environmental exposure to As is generally in the form of either arsenite (As^3+^) or arsenate (As^5+^) [6,8,9], with As^3+^ being more potent in its biological effects than As^5+^ [9,10]. As^3+^ is the predominant form in drinking water from deep (anaerobic) wells while As^5+^ predominates under aerobic conditions. Any As^5+^ in the water is rapidly reduced to As^3+^ as it enters cells [8,9]. Since As^3+^ displays high affinity for -SH groups perturbing essential proteins [11,12], As^3+^ compounds are of major human health concern. However, the precise mechanisms leading to lung damages and cancinogenesis by As^3+^ remain unclear and should be further investigated. 

Microtubules (MTs) are a highly organized network of filamentous structures in the cytoplasm of eukaryotic cells [13]. The principal protein of MTs is a heterodimer consisting of tightly linked globular peptides, *i.e.*, α- and β-tubulins, which polymerize into long, hollow, cylindrical filaments within the cell. In addition, a third subunit of MTs is γ-tubulin, functioning in the nucleation of mitotic spindle MTs. The most prominent feature of MTs is a rapid exchange between tubulin subunits and polymers. Lung cells contain abundant MTs which play a critical role in maintaining cellular architecture and internal organization, and in regulating cell shape, motility, transport, division, *etc*. [14]. Many drugs and toxicants can interact with cytoskeletal MTs [13,15]. For example, colchicine and vinblastine disassemble MTs and block mitotic spindle formation. In contrast, taxol promotes tubulin assembly and stabilizes MTs. Notably, metal ions display important modulating effects on MT dynamic. As shown by us and others, Mg^2+^, Be^2+^ and Ni^2+^ promote MT assembly while Ca^2+^, Cu^2+^, Cd^2+^, Hg^2+^, Cr^6+^ and As^3+^ cause MT disassembly [16,17,18,19,20,21]. Thus, MT injuries may be a critical manifestation of the pathogenesis of a number of toxic agents. 

Our previous studies have shown As^3+^ disassembly of MTs in cultured Swiss 3T3 cells [21]. To further understand mechanisms for cellular toxicity of As in the lung, we examined effects of As^3+^, a major toxic form of As in the nature, on MT assembly *in vitro*, in cultured rat lung fibroblasts (RFL6) and in lung tissues of the rat animal model. Results demonstrated that As^3+^ targets tubulin sulfhydryls and MT-associated proteins (MAPs) disrupting the MT organization in tubulin polymerization *in vitro* and in rat lung cells *in vitro* and *in vivo*.

## 2. Materials and Methods

### 2.1. Materials

Sodium *m*-arsenite (As^3+^, NaAsO_2_, analytical grade with purity ≥ 99.0%), dithiothreitol (DTT), N-ethylmaleimide (NEM), propidium iodine, and taxol were from Sigma-Aldrich Co (St. Louis, MO, USA). As^3+^ stock solutions were freshly prepared for each experiment. A rabbit anti-tubulin antibody and an FITC conjugated anti-tubulin antibody were from Polysciences (Warrington, PA, USA). A mouse anti-metallothionein antibody (Dako-MT, E9) against both metallothionein-I and metallothionein-II were from Dako Inc. (Fort Collins, CO, USA). A rabbit antibody against the heavy chain of γ-glutamylcysteine synthetase (γ-GCS) was from NeoMarkers (Fremont, CA, USA). An FITC-conjugated goat-anti-rabbit IgG was from Organon-Teknika (Malvern, PA, USA). A rabbit anti-actin (I-19) antibody and a horseradish peroxidase (HRP)-conjugated goat anti rabbit or mouse IgG were from Santa Cruz Biotech. (Santa Cruz, CA, USA). [^3^H]NEM (30–60 Ci/mmol) and [^35^S]methionine (1,000 Ci/mmol) were purchased from PerkinElmer Life and Analytical Sciences, Inc. (Waltham, MA, USA). Oligonucleotide primers for the PCR were synthesized by Integrated DNA Technologies (Coralville, IA, USA). Protein assay reagents for the Bradford dye binding method were from Bio-Rad (Richmond, CA, USA). All tissue culture products were from Invitrogen Co. (Carlsbad, CA, USA).

### 2.2. Cell Cultures

The fibroblastic RFL6 cell line derived from the fetal lung tissue of normal Sprague-Dawley rats was obtained from ATCC. Cells were regularly maintained in Dulbecco’s modified Eagle’s medium (DMEM) containing 10% fetal bovine serum (FBS). To standardize experimental conditions, cells were growth-arrested and synchronized at the G1 phase by incubation in 0.3% FBS/DMEM for 72 h followed by changing to fresh 0.3% FBS/DMEM and incubation for another 24 h before experiments [21]. Cells were treated for 24 h with As^3+^ or other reagents at various concentrations, then processed for further analysis. 

### 2.3. Fluorescence Microscopy

MTs were revealed fluorescently in control and treated cells with the primary rabbit anti-tubulin antibody and the secondary goat-anti-rabbit IgG coupled with FITC as described [22]. To assess As^3+^ effects on cell division, cells on coverslips were incubated in 10% FBS medium without or with 10 µM As^3+^ for 24 h. After fixation with 3.7% formaldehyde/0.2% Triton X-100/PBS and washing with PBS, cells were treated with an RNase A/PBS solution (the final concentration = 50 µg/mL) to lyse cellular RNA. Cell division stages and aberrations were visualized by the *in situ* chromosomes staining with propidium iodine (the final concentration = 50 µg/mL in PBS containing 2 mM MgCl_2_) and spindle MT staining with FITC-conjugated anti-tubulin antibody under the dark condition. Samples were examined under the Nikon fluorescence microscope using the DAPI-FITC-TRITC filter to detect green and red fluorescence simultaneously. All photographs were taken at the same magnification with a 40 × Planapochromat objective.

### 2.4. Immunohistochemistry and Total RNA Extraction in Lung Tissues of the Rat Animal Model

To assess As^3+^ injury to the lung MTs, eight Sprague-Dawley rats (body weight ≈ 150 g) per group were intratracheally instilled with 520–530 µg NaAsO_2_ in 100 µL physiological saline according to 2.02 mg As/kg body weight once a week for 5 weeks. Control rats received saline only. Rats were killed 1 week after the last instillation. For immunohistochemistry, lungs removed from four rats of each group were fixed with 0.2% glutaraldehyde and 4% paraformaldehyde in 0.1 M phosphate buffer, pH 7.4. Lung tissues were embedded in paraffin. Sections of 5 μm in thickness were immunohistochemically stained to visualize tubulin distribution in lungs with the anti-tubulin antibody and the streptavidin-HRP system according to the procedure provided by the manufacturer (KPL, Inc., Gaithersburg, MD, USA). For total RNA extraction, lungs in other four rats of each group were perfused with physiological saline via the pulmonary artery. The minced lung tissues were homogenized in TRIzol reagent (Invitrogen) and total RNA were extracted with phenol-chloroform as described [23].

### 2.5. Purification of MT Proteins

MT proteins containing tubulins and MAPs were purified from calf brain through two cycles of temperature-dependent assembly-disassembly as described in our previous publications [16,17]. The MT protein pellet was dissolved in a PME buffer (0.1 M Pipes, pH 6.6, 1 mM MgCl_2_ and 1 mM EGTA) and aliquots of this MT protein stock were stored at −80 °C until their use in experiments. Pure tubulin free of MAP was prepared by passing the twice-cycled MT proteins through a Whatman P11 phosphocellulose column as described [24].

### 2.6. Turbidity Assay

The original MT protein stock was diluted with the 0.1 M Pipes buffer, pH 6.6, to yield a final concentration of 0.8 mg/mL with 0.15 mM Mg^2+^ and 0.15 mM EGTA. MT polymerization *in vitro* was started by the addition of 500 µM GTP and monitored by turbidimetry at A_350 nm_ at 25 °C using a Perkin-Elmer Lambda 3B spectrophotometer equipped with a chart recorder [16,17]. To assess effects of As^3+^ on MT assembly *in vitro*, various concentrations of As^3+^ were added to the reaction mixtures for 5 min prior to addition of GTP. To observe DTT influence on As^3+^-induced effects on MT polymerization, As^3+^, DTT (1 mM) and final GTP were added to the reaction mixture in this order with the 3-min interval between additions to allow the interaction of As^3+^ with MT proteins. 

### 2.7. Electron Microscopy (EM)

EM examination of assembled MT polymers was carried out in conjunction with turbidity assays as described [16]. When the MT assembly reaction was approaching its plateau, a 10 µL-drop of the reaction mixture was removed from the cuvette and placed on glow-discharged carbon formvar-coated copper grids (300 mesh) for five min and successively replaced by cytochrome *c* (1 mg/mL, from Sigma) and distilled water. The samples were stained with filtered 1% uranyl acetate for 3 min, blotted, air dried, and examined with a Philips CM12 transmission electron microscope. All EM images were recorded on SO-163 film. MT numbers on three photo prints with the same size and magnification were counted for each sample and results are expressed as % of the control.

### 2.8. Tubulin Sulfhydryl (-SH) Assay

Tubulin -SH groups were determined as described in our previous publication [22]. This assay is based on covalent incorporation of [^3^H]NEM, a specific -SH group binding agent, to protein -SH groups. To quantitate As^3+^ effects on [^3^H]NEM binding to tubulin -SH groups, tubulin proteins free of MAPs prepared from the bovine brain were diluted with 10 mM phosphate buffer containing 0.3% NP40 to a final concentration of 1.5 mg/mL, pretreated with As^3+^ at indicated concentrations for 1 h at 0 °C, then mixed with [^3^H]NEM (2 µCi/mL), and incubated for an additional 1 h at 37 °C. Proteins were precipitated with 5% TCA and collected on nitrocellulose filters. Collected proteins on the membrane were measured by β-counting. The amount of radioactivity was normalized to total tubulin proteins and expressed as % of the control. Differences between control and As^3+^ treated samples (n = 3 for each group) were evaluated by using the ANOVA program as described [22]. 

### 2.9. Cytoskeletal and Cytosolic Protein Isolation in Cultured Cells

The cytoskeletal and cytosolic protein fractions in cultured cells were prepared by detergent extraction and centrifugation as described [21]. Briefly, control and treated cells were rinsed with PBS, and gently scraped into the MT stabilizing buffer (PM2G) containing 0.1 M Pipes, pH 6.9, 1 mM MgSO_4_, 2 M glycerol, and 2 mM EGTA, and extracted with 0.3% NP-40 in the PM2G buffer. After micro-centrifugation, the supernatant was collected and the pellet resuspended in a PM buffer (0.1 M Pipes, pH 6.9, 1 mM MgSO_4_) followed by centrifugation. The supernatant was mixed with the first extract and referred to as the “cytosolic” fraction. The final pellet was resuspended in 0.3% NP-40 in 10 mM phosphate buffer (pH 7.0) containing 1 mM EGTA and termed as the “cytoskeletal” fraction. Total protein concentrations in each fraction were determined by using the reagent from Bio-Rad as described [22]. 

### 2.10. Western Blot Analysis

Cytosolic and cytoskeletal lysates or total cell extracts in control and treated cells each containing equal amounts of protein (10–15 µg) were boiled in an SDS sample buffer and analyzed by SDS-PAGE gradient gel (4–15%) as described [23] using primary antibodies, *i.e.*, the rabbit anti-tubulin (1:1,000), the mouse anti-metallothionein (1:500), the rabbit anti-γ-GCS (1:1,000), or the rabbit anti-actin (1:1,000) antibody, and HRP conjugated corresponding secondary antibodies, *i.e.*, the anti-rabbit or mouse IgG with 1:5,000 dilutions. Molecular weights were determined by comparison with BenchMark pre-stained protein ladder (Invitrogen). Protein bands were quantitated by the 1 D Scan EX software (Scananalytics, Fairfax, VA, USA). Experiments as shown here were repeated at least three times with reproducible results and a representative one is presented, unless otherwise indicated. 

### 2.11. 2-D Gel Analysis

Cell metabolic labeling, cytoskeletal protein extraction and 2-D gel analysis were carried out as described [25]. Briefly, growth-arrested cells were incubated with [^35^S]methionine at 50 µCi/mL for 24 h in methionine-free DMEM in the presence or absence of 10 μM As^3+^. Cells treated with 5 µg/mL nocodazole, an MT depolymerization agent, at the last 3 h incubation, were used as a positive control. Cells were washed with PBS followed by PM2G with the protease inhibitor cocktail (Roche, Mannheim, Germany), extracted twice with 0.3% NP-40 in PM2G containing protease inhibitors and finally with 5 mM CaCl_2_ (Ca^2+^). Ca^2+^ released MT proteins including tubulins and MAPs were used for 2-D gel analysis as described [25]. Isoelectric focusing gels (3% acrylamide) with gradients of approximately pH 4.5–7.5 were obtained with 0.44% pH 3.5–10 Ampholines and 1.76% pH 6–8 Ampholines (LKB). The pH gradient was determined by measuring the pH value of 1-cm slices of parallel isoelectric focusing gels following overnight-incubation in 0.025 M KCl. The second dimension gels using 7.5% acrylamide were run with molecular markers (Bio-Rad) and stained with 0.25% Coomassie Brilliant Blue. Gels were treated with EN3HANCE (New England Nuclear Co., Boston, MA, USA), dried and exposed to prefogged Kodak X-Omat AR film at –80 °C. Protein spots were quantitated by the 1 D Scan EX software (Scananalytics) as described [23].

### 2.12. Reverse Transcription-Polymerase Chain Reaction (RT-PCR)

Total RNA (500 ng) extracted from cultured cells or from lung tissues were converted to the cDNA which was then amplified by using the SuperScript One-Step RT-PCR with Platinum *Taq* Kit (Invitrogen) under the following conditions: reverse-transcript at 50 °C for 30 min and pre-denaturation at 94 °C for 2 min, denaturation at 94 °C for 30 s, annealing at 58 °C for 45 s, and extension at 72 °C for 1 min for a total of 35 repetitive cycles. Final extension was performed at 72 °C for 10 min. The primer pair used to amplify βI-tubulin DNA is: forward, 5’-ATGAGGGAAATCGTG CACATCCAG-3’ and reverse, 5’-TTAGGCCTCTTCTTCTGCCTCC-3’. The primer pair used to amplify glyceradehyde 3-phosphate dehydrogenase (GAPDH) DNA, an internal control, is: forward, 5’-GACTCTACCCACGGCAA-3’ and reverse, 5’-GGATGACCTTGCCCACA-3’. PCR products were respectively separated on agarose gels, stained with ethidium bromide and visualized on a UV transilluminator. Bands of cDNA were quantitated with 1 D Scan analysis [23].

## 3. Results

**Figure 1:**
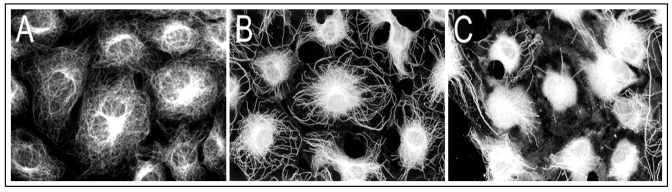
As^3+^ induced disassembly of MTs in RFL6 cells. Growth-arrested RFL6 cells on coverslips were treated without or with As^3+^ at indicated concentrations for 24 h, and then immunofluorescently stained for visualization of MTs. (**A**) control; (**B**) 5 µM As^3+^; (**C**) 20 µM As^3+^.

To elucidate As^3+^ effects on cellular MT organization, growth arrested RFL6 cells on coverslips were incubated with or without freshly prepared As^3+^ solution at final concentrations as indicated. Cellular MT distribution was revealed by immunocytochemistry [21]. As shown in Figure 1(A), growth-arrested RFL6 cells contain an intricate, highly organized network of MTs that radiate outward from the MT organizing center (MTOC) in the perinuclear region to the cell periphery. Exposure of cells to 5 µM As^3+^ for 24 h induced partial loss of MTs and no-organized tubulins with brightly staining accumulated in the perinuclear area where the MTOC is generally located [Figure 1(B)]. Treatment of cells with 20 µM As^3+^ for 24 h resulted in severe loss of MTs. Instead, mast cells exhibited high-density of depolymerized tubulin mass concentrated in the central area of cells [Figure 1(C)]. Notably, the maximum doses of As^3+^ (20 µM) that disrupted the MT organization in the cell model did not affect cell viability seriously (about 10% decrease) as assayed by trypan blue staining (data not shown). Morphological alterations in MTs were reversible after removal of As^3+^. These studies indicated that the MT cytoskeleton is an important cellular target for As^3+^ insult. 

**Figure 2:**
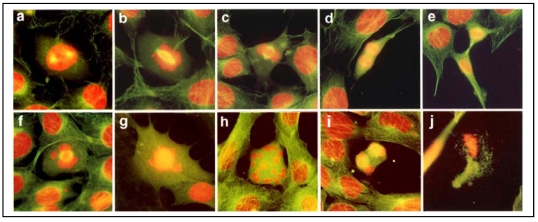
Deficient spindle MTs and cell division aberrations in As^3+^-treated cells. RFL6 cells on coverslips were incubated in 10% FBS medium without (**a–e**) or with (**f–j**) 10 µM As^3+^ for 24 h. Cell division stages and aberrations were examined by visualization of the *in situ* chromosomes stained with propidium iodine (red) and spindle MT stained with FITC-conjugated anti-tubulin antibody (green). **Note:** Cytoplasmic FITC-labeled MTs are green; however, since spindle MTs overlap with DNA staining in red, they appear as yellow structures. **a**, prophase; **b**, prometaphase; **c**, metaphase; **d**, anaphase; **e**, telophase; **f**, chromosome lagging with a defective spindle at metaphase; **g**, chromosome scattering in the absence of spindle; **h**, chromosome clustering and scattering in the absence of spindle; **i**, chromosome lagging with a defective spindle at anaphase; **j**, apoptosis of a cell with aberrant division.

Since spindle MTs are intrinsically involved in chromosomal organization during cell mitosis [13,14], As^3+^ may perturb spindle MTs inducing abnormal cell division. To test this, we have examined cell division aberrations by fluorescently double-staining spindle MTs and chromosomes *in situ* in As^3+^-treated RFL6 cells. As shown (Figure 2), exposure of RFL6 cells to 10 µM As^3+^ for 24 h resulted in the defect in or the absence of spindle MTs in 34% of proliferating cells (200 cells counted) accompanied by chromosomal lagging, scattering, clustering, *etc*., in 22% of proliferating cells (200 cells counted) [Figure 2(f–j)], in sharp contrast to those in control cells (only 2% of proliferating cells counted with spindle deficiency and 1.5% of proliferating cells counted with chromosomal disorientations). Figure 2(a–e) show normal chromosome patterns in different phases of the cell division. These results indicate that chromosomal disorientations are closely linked to As^3+^-induced damage to spindle MTs. 

**Figure 3:**
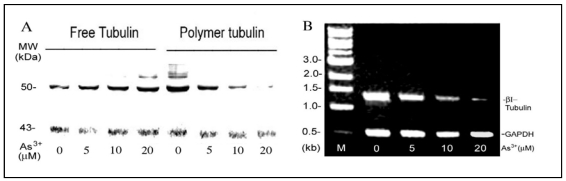
As^3+^ effects on expressions of tubulin proteins and mRNAs in the cell model. (**A**) As^3+^ enhanced the free tubulin pool as a result of depolymerization of MTs. Growth-arrested RFL6 cells were treated with As^3+^ at indicated concentrations for 24 h. Cytoskeletal and cytosolic fractions were isolated, and analyzed by Western blot. β-tubulin, 50 kDa; actin, 43 kDa used as a reference. (**B**) As^3+^ inhibition of steady-state βI tubulin mRNA levels. Cells were treated with As^3+^ as indicated above. Total RNA extracted was assessed for steady-state βI tubulin mRNA levels by the RT-PCR. GAPDH mRNA levels were used as an internal control. M indicates a DNA molecular ladder.

As^3+^-induced MT disassembly (Figure 1) should decrease the polymer MT and increase the free tubulin pool. To test this, As^3+^-treated cells were treated with 0.3% NP40 in the PM2G buffer to isolate cytosolic and detergent-resistant cytoskeleton fractions [21]. Western blot analysis indicated that in comparison to the actin control, RFL6 cells exposed to As^3+^ exhibited a dose-dependent decrease in polymer MT proteins and increase in the free tubulin pool [Figure 3(A)]. The density assays indicated that the 50% decrease in MT polymer proteins and increase in the free tubulin pool occurred in cells exposed to 5 µM As^3+^. These results are consistent with morphological loss of MTs and increase of free tubulin mass in these cells as revealed by fluorescent microscopy. Five β-tubulin genes encode highly homologous proteins, class I–V [26]. The rat class I β-tubulin (βI-tubulin) gene was selected as a sample to elucidate As^3+^ modulation of tubulin transcription. As shown by the RT-PCR assay [Figure 3(B)], the steady-state βI tubulin mRNA levels in cells treated with 5, 10 and 20 µM As^3+^ were reduced to 66%, 26% and 6% of the control, respectively. Thus, As^3+^ depolymerization of MTs was associated with inhibition of tubulin mRNAs.

**Figure 4:**
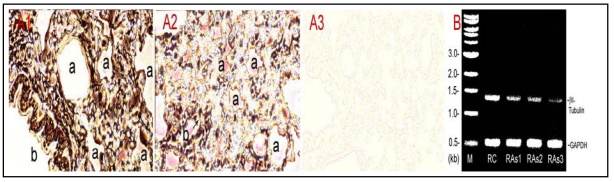
Inhibition of β-tubulin expression at protein and mRNA levels in As^3+^-exposed lungs. Rats (≈150 g body weight) were intratracheally instilled with 520–530 µg NaAsO_2_ in 100 µL physiological saline according to 2.02 mg As/kg body weight once a week for 5 weeks. Control rats received saline only. Rats were killed a week after the last instillation. Lung tissues were processed for immunohistochemical staining and total RNA extraction. (**A**) β-Tubulin protein distribution. The tubulin protein in control and As^3+^-dosed lung sections was stained with the antibody against β-tubulin and the streptavidin-HRP system. (**A1**) control; (**A2**) As^3+^ exposed lung; (**A3**) a negative control without the primary antibody treatment. a, alveolus; b, bronchioles; arrow indicates discontinuity of tubulin-rich epithelial cells in small bronchioles. (**B**) βI-tubulin mRNA levels revealed by the RT-PCR. RC, control rat; RAs1, As^3+^ treated Rat#1; RAs2, As^3+^-treated Rat#2; RAs3, As^3+^-treated Rat#3 (note: single doses for Rat #1, #2 and #3 were 520, 525 and 530 µg NaAsO_2_ , respectively, dependent on the body weight). M, a DNA molecular ladder.

To elucidate As^3+^ damage to the lung MTs, a small animal model study was performed to visualize tubulin protein distribution by immunohistochemistry and to assay for steady-state βI tubulin mRNA expression by the RT-PCR in control and As^3+^-dosed lungs. As shown [Figure 4(A)], immunoperoxidase staining of the lung tissue indicated markedly weakened tubulin signals, *i.e.*, peroxidase-labeled brown-black color materials, in As^3+^-treated lung tissues [Figure 4(A2)] in comparison to the control [Figure 4(A1)]. Figure 4(A3) is a negative control tissue slice from control rats, which was incubated in the absence of the primary antibody against β-tubulin. Notably, As^3+^ exposure apparently induced discontinuity of tubulin-rich epithelial cells in small bronchioles (arrow). Furthermore, RT-PCR assays indicated that As^3+^-dosed lungs displayed decreased βI tubulin mRNA levels amounting to 53%, 48% and 20% of the control in As^3+^-exposed Rat#1, Rat#2 and Rat#3, respectively [Figure 4(B)]. Thus, As^3+^ inhibited pulmonary tubulin expression at protein and mRNA levels *in vivo*.

**Figure 5:**
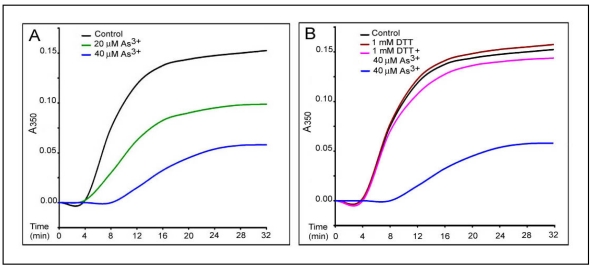
As^3+^-inhibition of MT protein polymerization *in vitro* as revealed by the turbidity assay. The *in vitro* polymerization of MT proteins (0.8 mg/mL) in 0.1 M Pipes buffer, pH 6.6, containing 0.15 mM Mg^2+^, 0.15 mM EGTA and 500 µM GTP was monitored by turbidity readings at A_350nm_ at 25 °C in the absence or presence of As^3+^ at indicated concentrations. Curves represent the time course of tubulin assembly under different conditions. (**A**) dose-dependent inhibition by As^3+^ of MT assembly. the black curve, control; the green curve, 20 µM As^3+^; the blue curve, 40 µM As^3+^. (**B**) DTT effects on As^3+^ inhibition of MT assembly. DTT (1 mM) was added after preincubation of polymerization mixture in the presence of As^3+^ for three min. the black curve, control; the brown curve, 1 mM DTT; the blue curve, 40 µM As^3+^; the pink curve, 40 µM As^3+^ plus 1 mM DTT.

**Figure 6:**
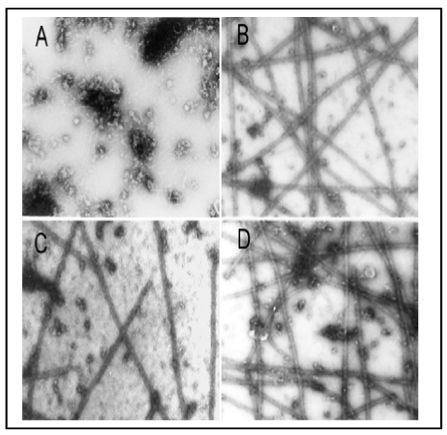
As^3+^-inhibition of MT protein polymerization *in vitro* as confirmed by EM examination. The *in vitro* MT polymerization was carried out as described in Figure 5. At the plateau level of the MT assembly (30 min after reaction), a 10 µL-drop of the reaction mixture was removed and processed for EM examination. (**A**) the absence of polymerized MTs at 0 min; (**B**) the typical morphology of *in vitro* assembled MTs at 30 min; (**C**) 40 µM As^3+^; (**D**) 40 µM As^3+^ plus 1 mM DTT. Photographs with a final magnification of 22,000 × observed under a Philips 300 transmission electron microscope.

To identify characteristics of the interaction of As^3+^ with tubulin proteins, we assessed its effects on the MT polymerization *in vitro*. Bovine brain MT proteins containing tubulins and MAPs were purified through two cycles of temperature dependent assembly and disassembly [17]. MT assembly *in vitro* was monitored by turbidity assays at A_350 nm_ (Figure 5) and confirmed by electron microscopy (EM) (Figure 6) as described [16,17]. In a typical turbidity assay at 25 °C [the black curve, in Figure 5(A)], assembly of MT proteins (0.8 mg/mL) progressed steadily with an initial leg (two min) followed by an elongation phase (2–30 min) and a final plateau indicating the reaction equilibrium (30–32 min). EM examination of samples taken at 30 min revealed the typical morphology of *in vitro* assembled MTs [Figure 6(B)]. No assembly of MTs was evident at time 0 [Figure 6(A)]. Incubation with As^3+^ induced a dose-dependent inhibition of MT assembly as evidenced by enhancement of the initial leg, decreases in the elongation rates and the plateau levels. For example, the average elongation rates at 12 min of the reaction (the turbidity values/time) were decreased to 50.0%, and 10.4% of the control at 20 (the green curve) and 40 (the blue curve) µM of As^3+^, respectively [Figure 5(A)]. The inhibition of MT assembly by As^3+^ was further confirmed by EM which showed significant reduction in the number of MT fragments in the presence of 40 µM As^3+^ [Figure 6(C)], amounting to 50% of the control [Figure 6(B)]. Importantly, addition of 1 mM dithiothreitol (DTT), a thiol reducing agent, to the reaction mixture strongly abolished the As^3+^ inhibitory effect on MT assembly as shown in the turbidity [the pink curve in Figure 5(B)] and EM assays [Figure 6(D)] reaching 95% (the turbidity assay) and 108% (the EM assay) of corresponding controls, respectively. Thus, As^3+^ inhibited MT polymerization *in vitro* and results provide a critical clue for the direct interaction of As^3+^ with tubulin -SH groups.

**Figure 7:**
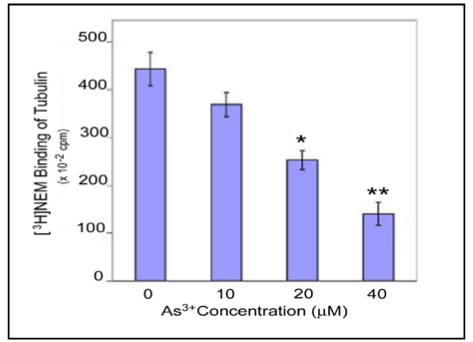
As^3+^ binding to tubulins through -SH groups as revealed by [^3^H]NEM binding assay. “Pure” tubulin free of MAP (1.5 mg/mL) prepared from bovine brain was pretreated with As^3+^ at indicated concentrations for 2 h at 0 °C, mixed with [^3^H]NEM (2 µCi/mL), and incubated for an additional 2 h at 37 °C. Proteins were precipitated with 5% TCA and filtered. Radioactivities coupled with collected proteins on the membrane were measured by β-counting. The significance of data presented was assessed by ANOVA analysis. * *p* < 0.05, ** *p* < 0.01 (n = 3).

To further assess As^3+^ binding to tubulin protein -SH groups, we also examined the competition of As^3+^ with N-ethylmaleimide (NEM), a sulfhyryl group reagent [21], for tubulin binding. “Pure” tubulin free of MAP (1.5 mg/mL) prepared from bovine brain was pretreated with As^3+^ at indicated doses for 2 h at 0 °C, mixed with [^3^H]NEM (2 µCi/mL), and incubated for an additional 2 h at 37 °C. Proteins were precipitated with 5% TCA and filtered. Radioactivities associated with collected proteins on the membrane were measured by β-counting. As shown (Figure 7), As^3+^ decreased the binding of [^3^H]NEM to tubulin with 50% inhibition occurring at 23 µM. This straightforward experiment shows As^3+^ binding to tubulin through -SH groups, initiating the cascade of MT disruption events.

**Figure 8:**
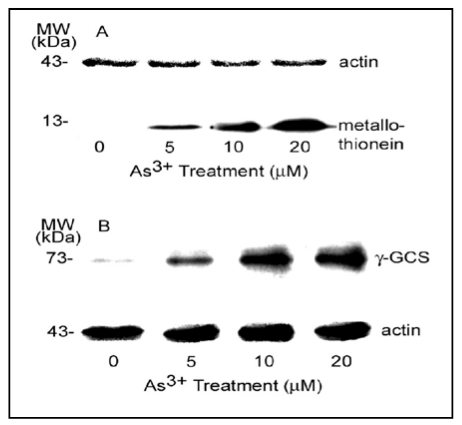
As^3+^ enhanced expression of metallothionein and γ-GCS. Growth-arrested RFL6 cells were treated with As^3+^ at indicated concentrations for 24 h and processed for Western blot analysis. (**A**) metallothionein (I and II); (**B**) γ**-**GCS heavy chain; actin as an internal control.

Cellular thiols such as glutathione (GSH) and metallothionein are important in maintaining the integrity of cytoskeletal structures and functions [21,22]. Interaction with cellular thiols is the most important biological property of As^3+^ [12,27]. Thus, we further examined the levels of other cellular thiols such as metallothionein (I and II isoforms) and thiol-related protein such as the heavy chain of γ-GCS, a rate limiting enzyme for GSH synthesis [28], in As^3+^-exposed RFL6 cells. As shown (Figure 8), treatment of cells with As^3+^ at indicated concentrations induced a dose-dependent elevation of cellular levels of metallothionein [Figure 8(A)] and the γ-GCS heavy chain [Figure 8(B)]. Thus, RFL6 cells exhibited a sensitive response to As^3+^ exposure in terms of altered cellular thiol homeostasis.

**Figure 9:**
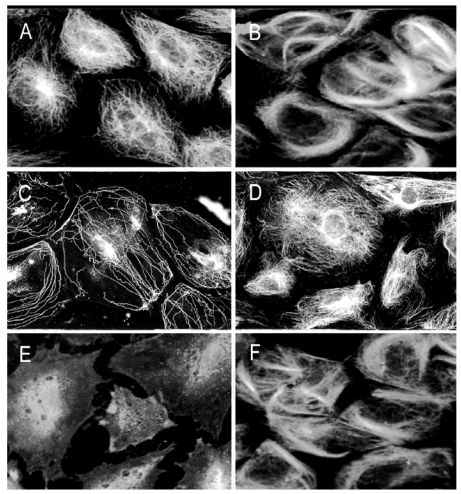
Taxol antagonistic effects on As^3+^-induced MT injury. Growth–arrested RFL6 cells on coverslips were treated with As^3+^, NEM, or their combinations with taxol for 24 h, and then stained with an anti-β tubulin antibody-FITC to visualize MTs. (**A**) control cells; (**B**) cells treated with 20 µM taxol; (**C**) cells treated with 20 µM As^3+^; (**D**) cells treated with 20 µM As^3+^ + 20 µM taxol; (**E**) cells treated with 25 µM NEM; (**F**) cells treated with 25 µM NEM + 20 µM taxol.

Taxol is an MT stabilizing drug [13]. To assess taxol protective effect on As^3+^-induced MT injury, we examined MT organization in As^3+^-treated RFL6 cells in the presence or absence of taxol. In comparison to the control [Figure 9(A)], treatment of cells with taxol at 20 µM induced MT bundling [Figure 9(B)]. As expected, 20 µM As^3+^ exposure for 24 h induced severe loss of cellular MTs [Figure 9(C)]. In contrast, co-incubation with 20 µM taxol prevented As^3+^-induced MT disruption [Figure 9(D)]. In addition, taxol also blocked disassembly of MTs induced by NEM (25 µM), a sulfhydryl group binding agent [21], which was used as a positive control in this experiment [comparing Figure 9(F), NEM + Taxol with Figure 9(E), NEM alone]. Thus, taxol antagonizes disassembly of MTs by thiol-bound agents such as As^3+^ and NEM.

**Figure 10:**
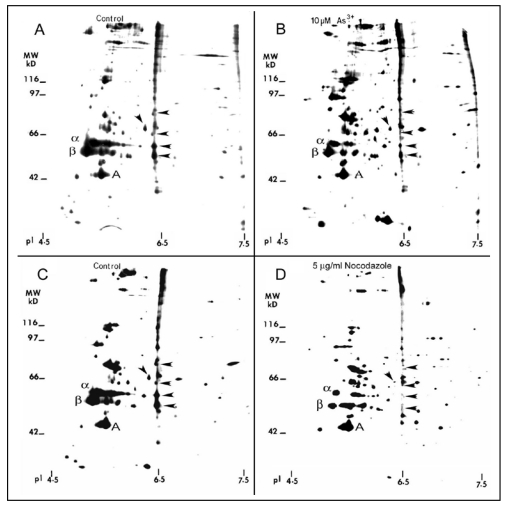
2-D gel profile of tubulins, MAPs, and other cytoskeletal proteins in As^3+^-treated cells. Growth-arrested RFL6 cells were labeled with [^35^S]methionine (50 μCi/mL) in the absence (**A**) or presence (**B**) of 10 µM As^3+^ for 24 h. Cells treated without (**C**) or with (**D**) 5 µg/mL nocodazole, an MT depolymerization agent, at the last 3 h of the metabolic labeling, were used as a positive control. The MT protein-rich extracts with identical redioactivity from control and treated cells were processed for 2-D gel analysis (α, β and A indicate α tubulin, β tubulin and actin, respectively). For MAP nomenclature, e.g., MAP56/6.5, the numbers refer to their apparent molecular weight (MW in kDa) and isoelectric point (pI), respectively. Protein quantitation was measured by the 1 D Scan EX software. Arrowheads indicate altered MAPs.

As^3+^ damage to the MT organization may be facilitated by alterations in MAPs which are important in maintaining the structural and functional integrity of MTs [29]. Because MTs are very sensitive to Ca^2+^, proteins released by Ca^2+^ extraction of the detergent resistant MTs of cells, by definition, contain tubulins and MAPs [25]. As analyzed by two-dimension (2-D) gels, quiescent RFL6 cells treated with 5 µg/mL nocodazole, a MT disassembly agent, a positive control in this study, for 3 h, displayed marked reduction of MT proteins in association with disappearing several MAPs such as 50/6.5, 53/6.5, 60/6.4, 62/6.3 and 86/6.5 (apparent MW in kDa/isoelectric point) indicated by arrowheads [compare Figure 10(D) with Figure 10(C)]. Similarly, cells incubated with 10 µM As^3+^ for 24 h exhibited a decrease in α and β tubulins and those MAPs sensitive to nocodazole [arrowheads in Figure 10(B)] in comparison to the control in the absence of As^3+^ treatment [Figure 10(A)]. Note that the internal control of actin was not significantly changed. Quantitative densitometric analysis indicated that the integrated intensities of MAPs 50/6.5, 53/6.5, 60/6.4, 62/6.3 and 86/6.5 were decreased to 44%, 49%, 15%, 48%, and 12% of the control, respectively. In view of the critical role of MAPs in the MT stability [29], decreases in the levels of several MAPs would contribute to MT instability favoring their disassembly. 

## 4. Discussion

Our previous studies have shown As^3+^ perturbation of the cytoskeletal organization and cellular glutathione homeostasis in Swiss 3T 3 cells [21]. The lung is a major target organ for arsenic. In this report, we further evaluated As^3+^ effects on the organization of MTs, a critical element of the cytoskeleton, *in vitro*, in cultured rat fetal lung fibroblasts and in lungs of the animal model. RFL6 cells were selected as an *in vitro* model in this study because they display a typical MT morphology sensitive to As^3+^ injury (Figure 1). Results indicated that As^3+^ disrupted MT assembly by targeting tubulin sulfhytryl groups and MAPs, two major stabilizers of MTs. Our findings showing As^3+^-elicited biochemical pathology of MTs are summarized in Table 1.

**Table 1:** Summary for As^3+^-elicited MT Biochemical Pathology.

Figure#	Stressor/antagonist	System	MT biochemical pathology by As^3+^
1	As^3+^ 5 µM, 20 µM.	MTs in RFL6 cells.	Disassembly of MTs.
2	As^3+^ 10 µM.	Spindle MTs and chromosomal patterns in RFL6 cells.	Spindle MT deficiency, cell division aberration, chromosome clustering, scattering and lagging.
3	As^3+^ 5 µM, 10 µM, 20 µM.	Tubulin proteins and mRNA in RFL6 cells.	Increasing free tubulin pool, decreasing polymer tubulins, inhibiting βI tubulin mRNA.
4	As^3+^ intratracheal instillation (2.02 mg As/kg body in the form of NaAsO_2_ in 100 μL physiological saline) once a week for 5 weeks.	β-tubulin protein and mRNA in the lung of rats.	Inhibition of lung β-tubulin protein and mRNA levels.
5	As^3+^ 20 µM, 40 µM, DTT 1 mM.	Bovine brain tubulin polymerization *in vitro.*	Inhibition of MT polymerization *in vitro* and DTT abolishing As^3+^ effects assessed by turbidimetry.
6	As^3+^ 40 µM, DTT 1 mM.	Bovine brain tubulin polymerization *in vitro.*	Inhibition of MT polymerization *in vitro* and DTT abolishing As^3+^ effects conformed by EM.
7	As^3+^ 10 µM, 20 µM, 40 µM, [^3^H]NEM (2 µCi/mL).	NEM binding to pure tubulins free of MAPs *in vitro.*	Blocking NEM binding to tubulins.
8	As^3+^ 5 µM, 10 µM, 20 µM.	Metallothionein and γ-GCS proteins in RFL6 cells.	Enhancement of γ-GCS and metallothionein expression.
9	As^3+^ 20 µM, NEM 25 µM, taxol 20 µM.	MTs in RFL6 cells.	Taxol antagonized As^3+^ and NEM-induced MT disassembly.
10	As^3+^ 10 µM, nocodazole 5 µg/mL.	Tubulins and MAPs in RFL6 cells.	Suppression of tubulin and MAP expression, nocodazole as a positive control.

Arsenic is a metalloid element existing in inorganic and organic forms and in different oxidative states (−3, 0, +3 and +5) which distribute in water, soil and air from nature and anthropogenic sources. High-level exposure to arsenic is involved in workers in metal smelting, wood preservation and semiconductor industries [1]. The acute inhalation of arsenic dust or fume resulted in gastrointestinal effects, and central and peripheral nervous disorders such as vomiting, diarrhea, bloody urine, anuria, shock, convulsion coma and death [1,30]. Chronic exposure to arsenic in humans, by inhalation and oral routes via contaminated water, food and air, are strongly associated with anemia, peripheral neuropathy, lesions and carcinogenesis in the lung, liver, bladder, skin, kidney, *etc.* [1]. As^3+^ is 60-times more toxic than As^5+^ [31]. As^3+^ has a high affinity for sulfhydryl groups in proteins, such as enzymes, receptors or coenzymes. Binding of As^3+^ to critical thiol groups of proteins may be important mechanism for As^3+^ toxicity [12,27]. Our previous and present studies showing As^3+^ depolymerization of MTs indicated that tubilin, a critical cell structure protein, is a target for As^3+^ insult.

A dimeric tubulin molecule contains 20 cysteine residues (12 for α and eight for β), most of which are biochemically accessible. Maintenance of certain tubulin -SH groups in the reduced form is crucial for MT polymerization since their loss induces MT disassembly [32,33]. One such “assembly-critical” -SH group, for example Cys_239_, has been identified in β-tubulin [33,34]. Cys_239_ mediates the lateral interaction of tubulin molecules required for MT assembly [33]. Thus, tubulin -SH groups are of a critical stabilizer for MTs. In this report, to identify the mechanism for As^3+^ depolymerization of MTs, we performed the *in vitro* MT polymerization assay using purified tubulins isolated from bovine brain. As^3+^ incubation with tubulin proteins inhibited MT assembly *in vitro* and DTT, a thiol-reducing agent, reversed the As^3+^ suppression of MT assembly (Figure 5 and Figure 6) supporting As^3+^ interaction with tubulin -SH groups. One should be noted. As^3+^ concentrations used in this *in vitro* MT assembly study were higher than those used in cell culture studies. This is most likely due to: (1) low concentrations of MT proteins in cultured cells, and (2) more abundant MAPs in the bovine brain tissue compared to non-neural cells, e.g., fibroblasts [35]. As^3+^ doses used in present studies, *i.e.*, 5–20 µM (0.374–1.498 ppm) for RFL6 cells, 10–40 µM (0.748–2.996 ppm) for *in vitro* tubulin polymerization assays, and 2.02 ppm for the rat animal model, are relevant to human exposure situation. The As content in the well drinking water of some areas in India reaches 3.4 ppm [36]. Furthermore, using the *in vitro* [^3^H]NEM binding competition assay (Figure 7) we demonstrated again As^3+^ binding to tubulin -SH groups. Decreases in [^3^H]NEM association with tubulins in the protein mixture preincubated with As^3+^ provided more direct evidence for As^3+^ targeting -SH groups of MT proteins initiating the MT injury cascade. More importantly, As exposure stimulates the formation of the reactive oxygen species (ROS) and activation of oxidative stress signaling such as the NF-*k*B pathway in the biological system [37] which act as an amplification system to further facilitate oxidation of sulfhydryl groups on tubulins and cell injuries. As reported, cells treated with hydrogen peroxide exhibited disruption of the MT cytoskeleton [38].

Our previous studies have shown As^3+^-induced a biphasic response in cellular glutathione (GSH), *i.e.*, an initial decrease followed by a full restoration and overshooting. Inhibition of cellular GSH by buthionine sulfoximine (BSO), an inhibitor of GSH biosynthesis, apparently increased cell sensitivity to As^3+^ toxicity [21]. Since As^3+^ targets cellular thiols we also examined other thiol-containing molecules such as metallothionein as well as γ-GCS, a rate limiting GSH synthetase [28], in cell response to As^3+^. In this report, we illustrated As^3+^ enhanced expression of metallothionein and γ-GCS at the protein levels accompanied by depolymerization of MT. The metallothionein molecule with 20 Cys residues can bind six As^3+^ while GSH binds As^3+^ to form As(SG)3 complex which favors transferring of As^3+^ to other thiol containing components [39,40]. Increased expression of γ-GCS as an upstream event is required for downstream enhancement of cellular GSH in cells treated with As^3+^ [21]. The metallothionein gene contains multiple copies of the metal responsive element (MRE) in its promoter region. MRE-binding transcription factor-1 (MTF-1), a zinc (Zn) finger protein, interacts with the MRE transactivating the metallothionein gene [41]. Zn also binds to tubulin proteins stimulating MT assembly *in vitro*. Tubulins isolated from brains of Zn-deficient animals showed an impaired ability for polymerization of MTs [42]. As^3+^ upregulated methallothioneins possibly by its binding to sulfhydryls of tubulins or other proteins releasing bound Zn ions that in turn enhance MTF-1 affinity for MRE containing genes such as metallothionein [41]. The NF-E2-related factor-2 (Nrf2) regulates antioxidant response element (ARE)-mediated expression of γ-GCS heavy subunit and other xenobiotic metabolizing enzymes. Nrf2 binds to the cysteine-rich keap1 protein in the actin filaments as an inactive form in the cytoplasm. Upon oxidative stress such as As^3+^ exposure, modification of cysteine residues in the Keap1 protein results in the Nrf2 release and nuclear localization for ARE binding and transactivation of the genes [43]. Elevation of cellular metallothionein and γ-GCS/GSH may be a critical detoxification mechanism against As^3+^ toxicity.

Tubulin heterodimer binds two GTP molecules, one exchangeable in β-tubulin and another one nonexchangeale in α-tubulin. Upon polymerization, GTP bound to β tubulin is hydrolyzed to GDP by intrinsic tubulin GTPase activity. The resulting GDP-tubulin exhibits a reduced affinity for its neighbors and undergoes disassembly [35]. Thus, MTs exhibit dynamic equilibrium, growing and shrinking by the addition or loss of tubulin dimers from the ends of protofilaments. As^3+^ has been reported to inactivate the GTP binding site on tubulins, thus inhibiting the MT assembly *in vitro* [44]. The hydrolysis of GTP to GDP in β-tubulin increases the curvature of protofilaments, destabilizing MTs [45]. This suggests that modulating the curvature status of MTs can change its stability. Taxol binding to the β-tubulin subunit, specifically to peptides 1–31, 217–231 and 297–293 [46], has been shown to straighten the GDP protofilament and slow down the transition of protofilaments from straight to a curved configuration [45]. Taxol stabilization of MTs has been reported to resist depolymerization by Ca^2+^, cold temperature, dilution, *etc.* [47]. Here, we go on to demonstrate taxol antagonism on disassembly of MTs by sulfhydryl-directed agents such as As^3+^ and NEM. Presumably, As^3+^ binding to tubulin -SH-groups may enhance the curvature of protofilaments, thus instabilizing MTs. 

In addition to the tubulin dimer, MTs also contain several minor proteins, *i.e.*, MAPs. The procedures for metabolic labeling, extraction with detergent and Ca^2+^, analysis of MAPs and other related proteins on 2-D gels have been used in different cells in response to different agents [25,48]. As shown in our studies, As^3+^ induced disassembly of MTs. Autoradiograms of proteins present in the MT fraction under As^3+^ treatment conditions were compared with its “control” profile derived from cells without As^3+^ treatment to identify subtle changes in protein molecular species. A protein that is present in cells with intact MT but changed or absent when intact MT no longer exist in such cells (such as nocodazole and As^3+^ treatments) fulfilling the operational definition of MAPs. Because of its high resolution, the quantitative 2-D gel electrophoresis has been the most powerful technique used for identifying unknown MAP proteins. With this 2-D gel system, several MAP proteins have been identified [25,48]. However, only five of them were decreased by >50% in cells exposed to 10 μM As^3+^ [Figure 10(B)], indicating that not all MAPs were equally susceptible to this trivalent metalloid element. MAPs exist only in minute amounts in non-neuronal cells. Based on their apparent MW and pI on 2-D gels, none of these MAPs (Figure 10) appeared to resemble any existing MAP reported in the literature. This is not surprising since most MAPs identified so far, such as MAP1, MAP2, MAP3, Tau family and 210 kD protein (MAP4), *etc.*, are from either the brain tissue or cells of neural origin [29,49,50,51]. Notably, several MAP, e.g., MAP-2 and Tau proteins isolated from the brain tissue, stimulate MT assembly and enhance MT stability both *in vitro* and *in vivo* [29,50]. Thus, results in giving for inhibition of MAPs by As^3+^ would lead to the enhancement of MT instability.

Immunohistochemical studies indicated severe loss of tubulin synthesis and distribution in As^3+^-dosed lungs [Figure 4(A2)]. Since absorbed As^3+^ is distributed throughout the body [5,6], all types of lung cells are targets for As^3+^ insult including interstitial fibroblasts. In view of MT functions, loss of MTs/tubulins, a cell structural protein, may be a critical basis for As-induced lung pathology such as bronchiectasis, bronchitis, COPD, *etc*. Synthesis of tubulin is subjected to a novel feedback autoregulation, *i.e.*, the level of the free tubulin pool directly controls the level of tubulin expression [52]. Thus, As^3+^ disrupts MTs increasing the free tubulin pool, as a result, inhibiting new tubulin synthesis. Our studies have shown As^3+^ downregulation of tubulin synthesis occuring at the mRNA level, *i.e.*, marked reduction of steady-state βI-tubulin expression in *in vitro* and *in vivo* models [Figure 3(B) and Figure 4(B)]. As reported, the binding of tubulin subunits to the nascent N-terminal tetrapeptide (Met-Arg-Glu-Ile) of β-tubulin triggers the adjacent ribosome to activate an RNase that degrades the polysome bound tubulin mRNA, inducing its destabilization [53]. In addition to its effects on tubulin mRNA stability, As^3+^ may also reduce tubulin mRNA initiation (synthesis) leading to low levels of steady-state tubulin mRNAs in lung cells and tissues. 

MTs play a fundamental role in regulation of cell division. Mitosis requires precise execution of a number of MT-based processes such as reduplication and splitting of the centrosome, assembly of a bipolar spindle, attachment of spindle MTs to chromosomes through kinetochores, tension generation on kinetochores by the dynamics of bipolar spindle MTs, and finally pulling each chromatid to one of the spindle poles [54]. An error in any step of such interdependent subcellular processes could perturb chromosomal organization, resulting in failure of mitosis. In this study we have observed cell division aberrations in the forms of chromosome lagging, scattering and clustering associated with spindle MT injury in the cell model following As^3+^ exposure (Figure 2). These characteristic chromosomal disorientations are also evident in cells treated with colchicine, an MT disassembly reagent [55]. Disassembly of all spindle MTs by As^3+^ may induce chromosomal scattering and clustering at metaphase while partial injure of spindle MT (such as disconnection of a few spindle fibers with chromosomes and/or monopole or multipoles, *etc*.) may lead to chromosomal lagging at anaphase/telophase. The latter appears to result from escaping the mitotic block (*i.e.*, passing through metaphase) as seen [Figure 2(i)]. Importantly, lagging chromosomes are believed to induce aneuploidy [56], which is in turn closely related to tumorigenesis [57]. Oxidation and methylation status of As displayed various effects on the mitotic apparatus [58]. As^3+^-resulted in production of aneuploid cells was associated with disruption of both the MT assembly and the spindle formation [59]. Thus, As^3+^-induced disassembly of spindle MTs and aberrations in the cell division have a critical biological significance for human tumor pathogenesis.

## 5. Conclusions

Briefly, present studies demonstrate two molecular targets of MTs for As^3+^ insult, *i.e.*, tubulin -SH groups and MAPs. As^3+ binding^ to tubulin -SH groups and inhibition of MAPs enhanced MT instability leading to its disassembly. Increase in the free tubulin pool by As^3+^ as a result of MT disassembly triggered an autoregulation mechanism inducing inhibition of tubulin synthesis at protein and mRNA levels and thus further aggravating lung MT injury, which was accompanied by activation of cellular metallothionein and γ-GCS, a critical thiol defense mechanism. As^3+^ damage to MTs was antagonized by taxol. Cytoskeletal MT injury by As^3+^ may be a critical basis for pulmonary pathology. These findings are expected to provide a piece of information for preventing As^3+^ cellular toxicity in the lung.

## References

[1] U.S. EPA (2011). Arsenic Compounds.

[2] Kapaj S., Peterson H., Liber K., Bhattacharya P. (2006). Human health effects from chronic arsenic poisoning—A review. J. Environ. Sci. Health Part A.

[3] Tapio S., Grosche B. (2006). Arsenic in the aetiology of cancer. Mutat. Res..

[4] EPHA (2012). World Facing Increasing Threat of Arsenic Poisoning.

[5] Dutkiewicz T. (1977). Experimental studies on arsenic absorption routes in rats. Environ. Health Perspect..

[6] Jones F.T. (2007). Invited review, a broad review of arsenic. Poultry Sci..

[7] Guha Mazumder D.N. (2007). Arsenic and non-malignant lung diseases. J Environ. Sci. Health Part A.

[8] Rossman T.G. (2003). Mechanism of arsenic carcinogenesis: An integrated approach. Mutat Res..

[9] Bertolero F., Pozzi G., Sabbioni E., Saffiott U. (1987). Cellular uptake and metabolic reduction of pentavalent to trivalent arsenic as determinants of cytotoxicity and morphological transformation. Carcinogenesis.

[10] Wang T.C., Jan K.Y., Wang A.S., Gurr J.R. (2007). Trivalent arsenicals induce lipid peroxidation, protein carbonylation, and oxidative DNA damage in human urothelial cells. Mutat. Res..

[11] Kitchin K.T., Wallace K. (2008). The role of protein binding of trivalent arsenicals in arsenic carcinogenesis and toxicity. J. Inorg. Biochem..

[12] Hughes M.F. (2002). Arsenic toxicity and potential mechanisms of action. Toxicol. Lett..

[13] Jordan M.A., Wilson L. (2004). Microtutules as a target for anticancer drugs. Nat. Rev. Cancer.

[14] Bershadsky A.D., Vasiliev J.M. (1988). Cytoskeleton.

[15] Stanton R.A., Gernert K.M., Nettles J.H., Aneja R. (2011). Drugs that target dynamic microtubules: A new molecular perspective. Med. Res. Rev..

[16] Li W., Zhao Y., Gantz D.L., Chou I.-N. (2003). Nickel (Ni^2+^) enhancement of microtubule assembly is dependent on GTP function. Toxicol. Appl. Pharmacol..

[17] Li W., Zhao Y., Chou I.-N. (1996). Mg^2+^ antagonism on Ni^2+^-induced changes in microtubule assembly and cellular thiol homeostasis. Toxicol. Appl. Pharmacol..

[18] Hamel E., Lin C.M., Kenney S., Skehan P., Vaughns J. (1992). Modulation of tubulin-nucleotide interaction by metal ions: comparison of beryllium with magnesium and initial studies with other cations. Arch. Biochem. Biophys..

[19] Li W., Zhao Y., Chou I.-N. (1992). Cytoskeletal injury induced by hexavalent chromate. Toxicol. In Vitro.

[20] Li W., Zhao Y., Chou I.-N. (1993). Alterations in cytoskeletal protein sulfhydryls and cellular glutathione in cultured cells exposed to cadmium and nickel ions. Toxicology.

[21] Li W., Chou I.-N. (1992). Effects of sodium arsenite on the cytoskeleton and cellular glutathione in cultured cells. Toxicol. Appl. Pharmacol..

[22] Li W., Kagan H.M., Chou I.-N. (1994). Alterations in cytoskeletal organization and homeostasis of cellular thiols in cadmium-resistant cells. Toxicol. Appl. Pharmacol..

[23] Chen L.-J., Zhao Y., Gao S., Chou I.-N., Toselli P., Stone P., Li W. (2005). Downregulation of lysyloxidase and upregulation of cellular thiols in rat fetal lung fibroblasts treated with cigarette smoke condensate. Toxicol. Sci..

[24] Murphy D.B., Heibsch R.R. (1979). Purification of microtubule protein from beef brain and comparison of the assembly requirements for neuronal microtubules isolated from beef and hog. Anal. Biochem..

[25] Shaw J.P., Chou I.-N., Anand B. (1988). Rapid phosphorylation of microtubule-associated proteins through distinct mitogenic pathways. J. Biol. Chem..

[26] Nogales E. (2001). Structural insight into microtubule function. Ann. Rev. Biophys. Biomol. Struct..

[27] Valko M., Morris H., Cronin M.T. (2005). Metals, toxicity and oxidative stress. Curr. Med. Chem..

[28] Yan N., Meister A. (1990). Amino acid sequence of rat kidney γ-glutamylcysteine synthetase. J. Biol. Chem..

[29] Olmsted J.B. (1986). Microtubule-associated proteins. Ann. Rev. Cell Biol..

[30] Gerhardsson L., Dahlgren E., Eriksson A., Lagerkvist B.E., Lundström J., Nordberg G.F. (1988). Fatal arsenic poisoning—A case report. Scand. J. Work Environ. Health.

[31] Ratnaike R.N. (2003). Acute and chronic arsenic toxicity. Postgrad. Med. J..

[32] Britto P.J., Knipling L., McPhie P., Wolff J. (2005). Thiol-disulphide interchange in tubulin: Kinetics and the effect on polymerization. Biochem. J..

[33] Luduena R.F., Roach M.C. (1991). Tubulinsulfhydryl groups as probes and targets for antimitotic and antimicrotubule agents. Pharmacol. Ther..

[34] Bai R.L., Lin C.M., Nguyen N.Y., Liu T.Y., Hamel E. (1989). Identification of the cysteine residue of beta-tubulin affected by the antimitotic agent 2,4-dichlorobenzyl thiocyanate, facilitated by separation of the protein subunits of tubulin by hydrophobic column chromatography. Biochemistry.

[35] Gelfand V.I., Bershadsky A.D. (1991). Microtubule dynamics: Mechanism, regulation, and function. Ann. Rev. Cell Biol..

[36] Guha Mazumder D.N. (2008). Chronic arsenic toxicity & human health. Indian J. Med. Res..

[37] Qian Y., Castranova V., Shi X. (2003). New perspectives in arsenic-induced cell signal transduction. J. Inorg. Biochem..

[38] Lee C.F., Liu C.Y., Hsieh R.H., Wei Y.H. (2005). Oxidative stress-induced depolymerization of microtubules and alteration of mitochondrial mass in human cells. Ann. NY Acad. Sci..

[39] Jiang G., Gong Z., Li X.-F., Cullen W.R., Le X.C. (2003). Interaction of trivalent arsenicals with metallothionein. Chem. Res. Toxicol..

[40] Scott N., Hatlelid K.M., Mackenzie N.E., Carter D.E. (1993). Reactions of arsenic (III) and arsenic (V) species with glutathione. Chem. Res. Toxicol..

[41] Giedroc D.P., Chen X., Apuy J.L. (2001). Metal response element (MRE)-binding transcription factor-1 (MTF-1): Structure, function, and regulation. Antioxid. Redox Signal..

[42] Hesketh J.E. (1982). Zinc-stimulated microtubule assembly and evidence for zinc binding to tubulin. Int. J. Biochem..

[43] Copple I.M., Goldring C.E., Kitteringham N., Park B.K. (2008). The Nrf2-Keap1 defence pathway: Role in protection against drug-induced toxicity. Toxicology.

[44] Li Y.M., Broome J.D. (1999). Arsenic targets tubulins to induce apoptosis in myeloid leukemia cells. Cancer Res..

[45] Elie-Caille C., Severin F., Helenius J., Howard J., Muller D.J. (2007). Straight GDP-tubulinprotofilaments form in the presence of taxol. Curr. Biol..

[46] Rao S., He L., Chakravarty S., Ojima I., Orr G.A., Horwitz S.B. (1999). Characterization of the taxol binding site on the microtubule. Identification of Arg(282) in beta-tubulin as the site of photoincorporation of a 7-benzophenone analogue of taxol. J. Biol. Chem..

[47] Schiff P.B., Fant J., Horwitz S.B. (1979). Promotion of microtubule assembly *in vitro* by taxol. Nature.

[48] Solomon F. (1986). Direct identification of microtubule-associated proteins by selective extraction of cultured cells. Methods Enzymol..

[49] Bloom G.S., Vallee R.B. (1983). Association of microtubule-associated protein 2 (MAPs) with microtubules and intermediate filaments in cultured brain cells. J. Cell Biol..

[50] Drubin D.G., Kirschner M.W. (1986). Tau protein function in living cells. J. Cell Biol..

[51] Ookata K., Hisanaga S., Bulinski J.C., Murofushi H., Aizawa H., Itoh T.J., Hotani H., Okumura E., Tachibana K. (1995). Cyclin B interaction with microtubule-associated protein 4 (MAP4) targets p34^cdc2^ kinase to microtubules and is a potential regulator of M-phase microtubule dynamics. J. Cell Biol..

[52] Cleveland D.W., Lopata M.A., Sherline P., Kirschner M.W. (1981). Unpolymerizedtubulin modulates the level of tubulin mRNAs. Cell.

[53] Claveland D.W. (1988). Autoregulated instability of tubulin mRNA: A novel eukaryotic regulatory mechanism. TIBS.

[54] Sharp D.J., Rogers G.C., Scholey J.M. (2000). Microtubule motors in mitosis. Nature.

[55] Warr T.J., Parry E.M., Parry J.M. (1993). A comparison of two *in vitro* mammalian cell cytogenetic assays for the detection of mitotic aneuploidy using 10 known or suspected aneugens. Mutat. Res..

[56] Natarajan A.T. (1993). An overview of the results of testing of known or suspected aneugens using mammalian cells *in vitro*. Mutat. Res..

[57] Chi Y.H., Jeang K.T. (2007). Aneuploidy and cancer. J. Cell Biochem..

[58] Kligerman A.D., Doerr C., Tennant A.H. (2005). Oxidation and methylation status determine the effects of arsenic on the mitotic apparatus. Mol. Cell. Biochem..

[59] Ramírez P., Eastmond D., Laclette J.P., Ostrosky-Wegman P. (1997). Disruption of microtubule assembly and spindle formation as a mechanism for the induction of aneuploid cells by sodium arsenite and vanadium pentoxide. Mutat. Res..

